# Latent variable modeling improves AKI risk factor identification and AKI prediction compared to traditional methods

**DOI:** 10.1186/s12882-017-0465-1

**Published:** 2017-02-08

**Authors:** Loren E. Smith, Derek K. Smith, Jeffrey D. Blume, Edward D. Siew, Frederic T. Billings

**Affiliations:** 1 Department of Anesthesiology, Vanderbilt University Medical Center, 1211 21st Avenue South, Nashville, TN 37205 USA; 2 Department of Biostatistics, Vanderbilt University Medical Center, Nashville, TN USA; 3 Department of Medicine, Vanderbilt University Medical Center, Nashville, TN USA; 4 Division of Nephrology and Hypertension, Vanderbilt Center for Kidney Disease and Integrated Program for AKI Research, Vanderbilt University Medical Center, Nashville, TN USA

**Keywords:** Acute kidney injury, Creatinine, Latent variable, Mixture model, Prediction, Risk factor

## Abstract

**Background:**

Acute kidney injury (AKI) is diagnosed based on postoperative serum creatinine change, but AKI models have not consistently performed well, in part due to the omission of clinically important but practically unmeasurable variables that affect creatinine. We hypothesized that a latent variable mixture model of postoperative serum creatinine change would partially account for these unmeasured factors and therefore increase power to identify risk factors of AKI and improve predictive accuracy.

**Methods:**

We constructed a two-component latent variable mixture model and a linear model using data from a prospective, 653-subject randomized clinical trial of AKI following cardiac surgery (NCT00791648) and included established AKI risk factors and covariates known to affect serum creatinine. We compared model fit, discrimination, power to detect AKI risk factors, and ability to predict AKI between the latent variable mixture model and the linear model.

**Results:**

The latent variable mixture model demonstrated superior fit (likelihood ratio of 6.68 × 10^71^) and enhanced discrimination (permutation test of Spearman’s correlation coefficients, *p* < 0.001) compared to the linear model. The latent variable mixture model was 94% (−13 to 1132%) more powerful (median [range]) at identifying risk factors than the linear model, and demonstrated increased ability to predict change in serum creatinine (relative mean square error reduction of 6.8%).

**Conclusions:**

A latent variable mixture model better fit a clinical cohort of cardiac surgery patients than a linear model, thus providing better assessment of the associations between risk factors of AKI and serum creatinine change and more accurate prediction of AKI. Incorporation of latent variable mixture modeling into AKI research will allow clinicians and investigators to account for clinically meaningful patient heterogeneity resulting from unmeasured variables, and therefore provide improved ability to examine risk factors, measure mechanisms and mediators of kidney injury, and more accurately predict AKI in clinical cohorts.

**Electronic supplementary material:**

The online version of this article (doi:10.1186/s12882-017-0465-1) contains supplementary material, which is available to authorized users.

## Background

The diagnosis of acute kidney injury (AKI) relies primarily on changes in serum creatinine concentrations (ΔSCr) [1–3]. Changes in serum creatinine, however, can be insensitive and nonspecific for renal injury [4, 5] due to unmeasured confounders such as changes in creatinine production, myocyte injury, intravenous fluid administration, and renal functional reserve [6, 7]. Many of these unmeasured confounders, also known as latent variables [8], represent an important source of patient heterogeneity with respect to how and if AKI manifests, but are clinically impractical or impossible to measure. Failure to account for factors like these decreases power to identify risk factors for AKI and hinders accurate prediction of postoperative AKI.

Latent variable mixture modeling improves the ability to assess the associations between independent variables and an outcome by accounting for the effect of a latent variable. The model uses measured covariates to empirically stratify a cohort into subpopulations of patients, represented by a latent variable, which are more homogenous than the total cohort. These subpopulations are represented by component models which can be combined to form a comprehensive model to represent the entire cohort [9]. We hypothesized that a latent variable mixture model would increase power to identify significant risk factors for AKI and improve accuracy in predicting a patient’s postoperative ΔSCr compared to a traditional linear model. To test this hypothesis, we built a traditional linear model and a two-component latent variable mixture model to predict ΔSCr in a well-phenotyped clinical trial of AKI following cardiac surgery and compared the models’ goodness-of-fit, power to identify established AKI risk factors, discrimination, and prediction of 48-h postoperative ΔSCr.

## Methods

### Patient sample

After Vanderbilt University Medical Center Institutional Review Board approval, we collected data from a 653-subject prospective clinical trial of perioperative statin use to prevent AKI following cardiac surgery (NCT00791648). The study was conducted according to the Declaration of Helsinki. Patients were eligible to participate in the trial if they were scheduled for elective coronary artery bypass grafting, valve surgery, or ascending aortic surgery requiring thoracotomy or sternotomy. Patients receiving preoperative renal replacement therapy, with liver dysfunction, acute coronary syndrome, pregnancy, current CYP3A4 inhibitor use, and a history of kidney transplant or statin intolerance were ineligible to participate. Six hundred fifty-three patients provided written informed consent. Thirty-eight patients were excluded for failing inclusion criteria or withdrew for personal reasons prior to study initiation, and one patient that completed the study received hemodialysis on postoperative day one and was excluded from 48-h ΔSCr model development since this patient’s ΔSCr no longer reflected renal injury or function. Thus 614 patients were included. No significant association between perioperative statin use and postoperative AKI was demonstrated in the clinical trial [10].

### Modeling AKI

We chose maximum ΔSCr from baseline to postoperative day 2 to model AKI because serum creatinine is the most common and best characterized marker of renal injury, a 48-h interval is consistent with current consensus guidelines for AKI diagnosis, and a continuous scale rather than a binomial threshold for AKI preserves the measurement of AKI severity and provides the best opportunity to ascertain differences between linear and latent variable mixture modeling techniques. Baseline serum creatinine concentration was defined as the most recent preoperative creatinine measurement and was measured in inpatients on the morning of surgery and within a week prior to surgery in outpatients. Postoperative serum creatinine concentrations were measured at 2:00 am daily throughout hospitalization.

We selected model covariates a priori based on established predictors of post-cardiac surgery AKI and factors known to affect serum creatinine production or dilution [6, 9, 11–14]. Including well-established risk factors for AKI facilitates comparison of each model’s ability to identify significant AKI risk factors for the prediction of ΔSCr. Selected covariates were identical for both the linear model and the latent variable mixture model and included age, body mass index (BMI), baseline glomerular filtration rate estimated using the CKD-EPI formula (eGFR) [15], baseline serum creatinine, age●baseline serum creatinine interaction term, baseline hematocrit, presence of diabetes, presence of hypertension, duration of surgery, baseline pulse pressure, volume of hydroxyethyl starch administered during surgery, volume of urine output during surgery, duration of cardiopulmonary bypass, duration of aortic cross clamp, maximum intraoperative arterial lactate concentration, and average intraoperative mean blood pressure adjusted for baseline mean blood pressure. Dataset completion was excellent (100% of all serum creatinine data were complete; >99% of all covariate data were complete).

### Model development

A linear model and a two-component latent variable mixture model were each fit to the maximum ΔSCr from baseline over the first 48 postoperative hours.

The latent variable mixture model is composed of two traditional linear models, known as component models. Each component model represents a subpopulation of patients formed by the latent variable. During fitting, the mixture model agnostically identifies two distinctive subpopulations based on covariate patterns with respect to observed 48-h postoperative ΔSCr. Given that there is uncertainty regarding individual patient subpopulation membership (i.e., subpopulation membership is determined by each patient’s unknown latent variable status, 0 or 1), a probability of being in each subpopulation is initially randomly assigned to each patient and then refined during the iterative model fitting process until convergence criteria are met. Therefore, at the conclusion of model fitting, a patient whose covariate pattern is very consistent with subpopulation 1, for example, may be assigned a 90% probability of subpopulation 1 membership and a 10% probability of subpopulation 2 membership. In this way, each patient’s data may contribute to both component models, improving overall model fit. Each component model represents a data-identified patient subpopulation. If two distinct subpopulations are not identified during the fitting process, the first component model would become identical to the traditional linear model and the coefficients for all the covariates of the second component model would be assigned a value of zero. After completion of model fitting, we developed a support vector machine algorithm to predict patient subpopulation allocation probabilities based on covariate patterns but independent of observed ΔSCr. This enables prediction of ΔSCr using the latent variable mixture model and allows us to compare ΔSCr prediction between latent variable mixture and linear models.

### Statistical analyses

Patient characteristics were summarized with the 50th (10th, 90th) percentiles for continuous variables and percentages for categorical variables. To evaluate the latent variable mixture model relative to the linear model, we compared model: 1) goodness-of-fit, 2) average power to identify established risk factors for AKI, 3) discrimination (ability to rank subjects in order of predicted ΔSCr), and 4) accuracy to predict maximum 48-h postoperative ΔSCr.

Goodness-of-fit was assessed with calibration plots and r^2^ calculations of predicted ΔSCr versus observed ΔSCr for the latent variable mixture and linear models. To account for the increased flexibility of the latent variable mixture model with respect to differential model fit, Bayesian Information Criteria (BIC) values were calculated for each model and compared using a relative likelihood calculation.

The ability to identify AKI risk factors was assessed using a *post hoc* calculation of each model’s power to identify established risk factors as significant. For this calculation, 5000 new datasets were generated from our original dataset using standard parametric bootstrapping techniques, and both the latent variable mixture model and the linear model were refit in each new dataset. This produced a set of new risk factor coefficients and associated *p*-values for each model. Using these sets of new model coefficients, individual risk factor identification power comparisons between the two models were performed, taking our original fitted model coefficients as the power calculations’ alternative hypotheses. A sign test was used to determine the significance of the power comparison between the two models. Additionally, quantile-quantile (Q-Q) plots were used to assess the normalcy of each model’s errors in order to compare each model’s covariate coefficient accuracy.

Model discrimination was evaluated with a permutation test of each model’s Spearman’s correlation coefficients between predicted and observed ΔSCr.

To evaluate prediction of maximum 48-h postoperative ΔSCr, we compared the average of the square of the difference between the predicted and true ΔSCr (i.e., mean squared error relative difference [(predicted ΔSCr – true ΔSCr)^2^]) [16].

Models were bootstrapped with 200 replicates to assess for over-fitting and provide internal validation. Statistical analyses were performed in R (version 3.2.0, R Foundation, http://www.r-project.org) and included pROC and flexmix packages.

## Results

### Subject characteristics and AKI

Six hundred fourteen patients comprised the study cohort. The cohort was primarily Caucasian, and one third of patients were female (Table 1). Half of the patients received coronary artery bypass surgery, two-thirds valve replacement or repair, and three quarters of surgeries were performed with the use of cardiopulmonary bypass. One hundred thirty five patients (22.1%) developed KDIGO AKI. One hundred and nineteen of these patients met the 0.3 mg/dL increase within 48-h criterion, 72 the 50% increase within 7-days criterion, and 60 both. Twenty-six patients (4.2% of the total cohort) developed KDIGO stage II or III AKI, 5 of whom required postoperative renal replacement therapy.

**Table 1:** Cohort characteristics

Characteristic	All subjects (*n* = 615)
Age, years	67 (50, 81)
Female	188 (30.6%)
African American	26 (4.2%)
Body mass index, kg/m^2^	27.7 (22.5, 36.9)
Medical history	
Hypertension	544 (88.5%)
Congestive heart failure	243 (39.5%)
Left ventricular ejection fraction, %	60 (35, 60)
Myocardial infarction	110 (17.9%)
Prior cardiac surgery	110 (17.9%)
Diabetes	202 (32.8%)
Current smoking	88 (14.3%)
Chronic obstructive pulmonary disease	64 (10.4%)
Peripheral vascular disease	170 (27.6%)
Preoperative medication use	
Statin	416 (67.6%)
ACE inhibitor	192 (31.2%)
Baseline laboratory data	
Creatinine, mg/dl	1.01 (0.74, 1.60)
eGFR, ml/min/1.73 m^2^	72.8 (38.5 96.7)
Hematocrit, %	34 (25, 43)
Perioperative atorvastatin treatment assignment	308 (50%)
Procedure characteristics	
CABG surgery	301 (48.9%)
Valve surgery	397 (64.6%)
Cardiopulmonary bypass use	435 (70.7%)
Cardiopulmonary bypass time, min	110.0 (0, 211.6)
Aortic cross clamp use	291 (47.3%)
Aortic cross clamp time, min	0 (0, 139.6)
Intraoperative fluids	
Intravenous crystalloid, mL	1600 (1000, 3000)
Intravenous hydroxyethyl starch, mL	0 (0, 0)^a^
Urine output, mL	430 (175, 946)
Arterial lactate, maximum intraoperative, mmol/L	1.7 (0.9, 3.8)
Length of surgery, hours	5.1 (3.6, 7.8)

^a^Only 59 of 615 patients received intravenous hydroxyethyl starch during surgery accounting for the low 10th, 50th, and 90th percentile values. *BP* blood pressure, *ACE* angiotensin converting enzyme, *eGFR* estimated glomerular filtration rate using CKD-Epi formula, *CABG* coronary artery bypass grafting

Binary characteristics are reported as *n* (%) and continuous characteristics as median (10th percentile, 90th percentile)

The median serum creatinine and eGFR at baseline were 1.01 mg/dl (10th, 90th percentile: 0.74, 1.60) and 73 ml/min/1.73 m^2^ (38, 97). The median maximum ΔSCr within 48 h of surgery was 0.07 (−0.13, 0.52) for the total cohort, 0.50 mg/dl (0.27, 1.04) in patients that developed KDIGO AKI, and 0.03 (−0.15, 0.20) in patients that did not develop AKI.

### Latent variable mixture model subpopulation assignments

**Fig. 1:**
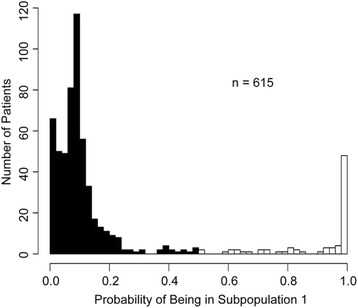
Histogram of patients’ probabilities of being a member of subpopulation 1 verses subpopulation 2, determined by a latent variable. Subpopulation 1 represents patients in whom AKI risk factors more strongly correlate with 48-h postoperative change in serum creatinine concentration, and subpopulation 2 less strongly, at completion of mixture model fit. The *black bars* represent patients with less than 50% probability of being a member of subpopulation 1 (>50% probability of being in subpopulation 2), and the *white bars* represent patients with greater than 50% probability of being a member of subpopulation 1 (<50% probability of being in subpopulation 2)

The two-component latent variable mixture model identified two distinct subpopulations of patients indicating the existence of a latent variable. At the completion of model fitting, 13% of patients had >50% probability of being in subpopulation 1, and 87% of patients had <50% probability of being in subpopulation 1 (i.e., 87% of patients had >50% probability of being in subpopulation 2 (Fig. 1)). If patients with a >50% probability of being in subpopulation 1 are assigned to subpopulation 1 and patients with >50% probability of being in subpopulation 2 are assigned to subpopulation 2, then in general subpopulation 1 tended to be older, with a greater prevalence of hypertension, diabetes, and congestive heart failure, and a lower baseline eGFR (Additional file 1: Table S1).

### Model fit

**Fig. 2:**
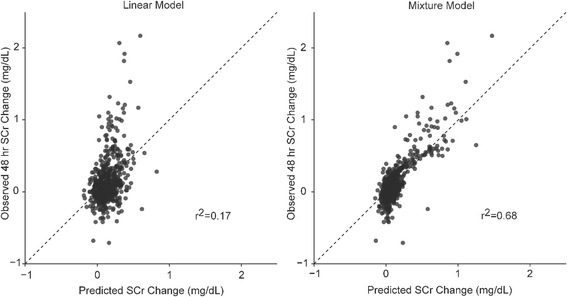
Calibration plots of the linear and latent variable mixture models’ predicted maximum change in serum creatinine concentration (ΔSCr) from baseline to 48-h after surgery versus the observed maximum ΔSCr from baseline to 48-h after surgery. The *dotted line* represents the line of best fit

The latent variable mixture model demonstrated superior goodness-of-fit throughout the range of predicted ΔSCr (Fig. 2), resulting in a BIC value of 140 for the latent variable mixture model compared to 349 for the linear model. These BIC values represent a 6.66 × 10^71^ times increased likelihood of the latent variable mixture model providing superior fit compared to the linear model.

### AKI risk factor identification and estimation accuracy

The latent variable mixture model identified a significant association between 14 of the 16 established AKI risk factors included as covariates and maximum 48-h ΔSCr, while the linear model demonstrated a significant association between 6 of the 16 established AKI risk factors and maximum 48-h ΔSCr (Table 2). *Post hoc* relative power calculations showed that the latent variable mixture model had greater power to identify established risk factors as significant for 15 of the 16 covariates considered compared to the linear model (sign test, *p* < 0.001). The latent variable mixture model exhibited 94% (−13 to 1132%) more power (median [range]) to identify established risk factors as having a statistically significant association with 48-h ΔSCr as the linear model.

**Table 2:** Associations between established AKI risk factor covariates and maximum 48-h serum creatinine change from baseline using a linear model and each subpopulation of a two-component latent variable mixture model

Risk factor	Linear model	Latent variable mixture model	
		Subpopulation 1	Subpopulation 2
Age (per 10 years)	0.037 (−0.044, 0.118)	0.040 (−0.021, 0.101)	0.042 (0.016, 0.068)**
BMI (per 5 kg/m^2^)	0.040 (0.013, 0.068)**	0.107 (0.081, 0.132)***	0.012 (0.005, 0.019)***
History of hypertension	0.005 (−0.046, 0.056)	0.080 (0.002, 0.158)*	−0.017 (−0.031, −0.003)*
History of diabetes	−0.024 (−0.082, 0.035)	−0.123 (−0.177, −0.068)***	−0.007 (−0.021, 0.008)
Baseline pulse pressure (per 10 mmHg)	0.003 (−0.009, 0.015)	−0.019 (−0.035, −0.003)*	0.004 (7.2e-5, 0.008)*
Baseline SCr (per mg/dL)	0.203 (−0.309, 0.715)	0.054 (−0.217, 0.326)	0.158 (−0.023, 0.339)
Baseline SCr:age interaction	−0.001 (−0.008, 0.007)	0.002 (−0.003, 0.007)	−0.001 (−0.003, 0.002)
Baseline eGFR (per 30 mL/min/1.73 m^2^)	0.081 (−0.012, 0.174)	0.045 (−0.066, 0.156)	0.099 (0.051, 0.147)***
Baseline hematocrit (per %)	−0.010 (−0.016, −0.005)***	−0.034 (−0.040, −0.028)***	−0.003 (−0.005, −0.001)***
Cardiopulmonary bypass time (per hour)	0.006 (−0.018, 0.030)	−0.072 (−0.108, −0.036)***	0.012 (2.0e-4, 0.024) **
Aortic cross clamp time (per hour)	0.036 (0.001, 0.072)*	0.156 (0.120, 0.192)***	−0.006 (−0.018, 0.006)
Intraoperative hydroxyethyl starch volume (per L)	0.200 (0.000, 0.400)	0.300 (0.100, 0.500)**	0.000 (−0.056, 0.094)
Intraoperative urine output (per L)	−0.100 (−0.200, −0.048)**	−0.300 (−0.400, −0.200)***	−0.100 (−0.094, −0.016)***
Mean intraoperative MAP adjusted for baseline MAP (per 10 mmHg)	0.023 (0.003, 0.042)*	0.064 (0.042, 0.086)***	0.005 (0.001, 0.009)*
Maximum intraoperative lactate (per mmol/L)	0.004 (−0.021, 0.028)	−0.009 (−0.033, 0.016)	0.013 (0.007, 0.019)***
Length of surgery (per hour)	0.034 (0.009, 0.059)**	0.113 (0.086, 0.140)***	0.027 (0.021, 0.034)***

*p < 0.05, **p < 0.01, ***p < 0.001; *BMI* body mass index, *eGFR* estimated glomerular filtration rate using CKD-Epi formula, *SCr* serum creatinine concentration, *MAP* mean arterial blood pressure

For example, an increase of ten years in age is associated with a 0.037 increase in 48-h postoperative change in serum creatinine concentration (ΔSCr) in the linear model, and a past medical history of hypertension was associated with a 0.080 increased in 48-h ΔSCr in the subpopulation 1 component model. Ninety-five percent confidence intervals are listed after each covariate coefficient estimate

**Fig. 3:**
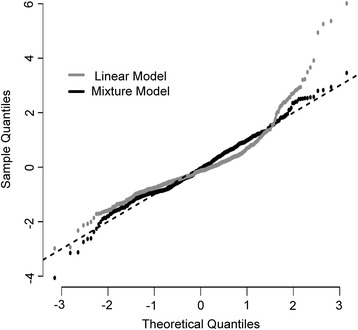
Quantile-quantile plot of the linear and latent variable mixture model’s error distributions. The *dotted line* represents the ideal distribution of model errors to ensure accurate risk factor identification

A Q-Q plot revealed that the latent variable mixture model deviated less from the line of best fit than the linear model (Fig. 3), demonstrating that the latent variable mixture model better fulfilled the linear regression requirement of normally distributed errors. This signifies that the latent variable mixture model has an improved ability to accurately assess associations between patient characteristics and postoperative ΔSCr compared to the linear model.

### Model discrimination and prediction of 48-h postoperative ΔSCr

The latent variable mixture model demonstrated superior discrimination for predicted ΔSCr compared to the linear model (Permutation test of Spearman’s correlation coefficients, *p* < 0.001). The relative mean squared error reduction for the latent variable mixture model comparative to the linear model was 6.8%, meaning that the latent variable mixture model predicted 48-h postoperative ΔSCr 6.8% more accurately.

## Discussion

In this study of perioperative AKI, a latent variable mixture model had markedly more power to identify established risk factors for AKI and improved ability to predict a patient’s postoperative ΔSCr than a traditional linear model. These benefits were likely due to superior goodness-of-fit, improved accuracy of covariate coefficient estimation, and enhanced discrimination of predicted postoperative ΔSCr. Latent variable mixture modeling may offer substantial benefits to the study of AKI, and future studies that seek to isolate risk factors for AKI, measure mechanisms of AKI, test therapies for AKI, or seek to predict AKI in clinical cohorts should consider using this methodology.

The improvement of AKI modeling with the latent variable mixture modeling technique indicates that substantial heterogeneity exists within the perioperative AKI population that is not accounted for by observed covariates, and that reliance on traditional linear modeling techniques which inherently assume observed covariates are the only relevant covariates obscures this heterogeneity. This unaccounted for patient heterogeneity within the AKI population may explain why numerous AKI prevention and intervention trials have failed to demonstrate efficacy despite promising preclinical trials.

While new to studies of AKI, latent variable mixture modeling is an established statistical methodology to account for patient heterogeneity in other clinical domains. It has long been used in psychology and genetics research [17, 18], and more recently in oncology. For example, the use of latent variable mixture modeling to model small cell lung cancer growth dynamics from serum biomarker data has improved the prediction of treatment outcomes and decreased reliance on sequential imaging [19]. In acute lung injury, a latent variable mixture modeling technique recently identified patient phenotypes associated with differential treatment effects of high versus low positive end expiratory pressure where traditional modeling had failed [20]. Identification of latent variable subpopulations in patients at risk for AKI may also lead to the identification of subpopulation-specific treatment benefits, enhanced risk stratification, and improved prediction of long-term outcomes.

In the current study, the latent variable mixture model displayed greater power to identify established risk factors for AKI. This improvement results in increased power to identify and characterize novel candidate risk factors, including baseline characteristics, intraoperative exposures, perioperative biomarkers, and patient management techniques that could be modified to reduce AKI. Candidate factors are easily evaluated using the latent variable mixture model by adding the candidate factor to both component models before mixture model fitting. The *p*-value associated with the candidate factor’s coefficient for each component model determines the significance of the candidate factor in each patient subpopulation. Using latent variable mixture modeling to assess candidate factors will increase discernment of their association with AKI and benefit the search for other non-latent, modifiable AKI risk factors, particularly in modestly sized patient cohorts where power may be low.

Development of the latent variable mixture model does not itself identify the latent variable or binomial pattern of variables, but can suggest potential candidates including renal functional reserve, genetic polymorphisms, clusters of disease exposure, fluid management strategies, or surgical treatments. For example, renal functional reserve is a potentially source of heterogeneity in susceptibility that leads to variation in the manifestation of AKI across patients [7, 21–26]. In our study, older age and higher comorbidity burden (e.g., diabetes, hypertension) is potentially consistent with a population with less renal reserve compared to subpopulation 2 in whom traditional risk modeling performed less well [7, 27, 28]. In the former, a potential lack of renal reserve might explain the larger model coefficients associated with established AKI risk factors such as history of hypertension and diabetes, BMI, baseline hematocrit, aortic cross clamp duration, and length of surgery. In contrast, the potential presence of renal functional reserve might contribute to smaller, and frequently statistically insignificant, model coefficients for established AKI risk factors in subpopulation 2. The latter subpopulation might represent patients in whom sensitive AKI biomarkers may better predict the potential long-term impact of AKI than currently emphasized risk factors. Irrespective of the identity of the latent variable, our results indicate that latent variable mixture modeling can identify subpopulations of patients that may be used to enrich outcomes in clinical trials, target monitoring and interventions, and shed novel insight into the pathophysiology of AKI.

Strengths of this study include the use of high-quality unbiased data collected as part of a prospective clinical trial with little to no missing data. We also retained serum creatinine as a continuous variable to enhance AKI discrimination and prediction [29, 30]. At the same time we acknowledge potential limitations. We did not evaluate latent variable mixture models with more than two subpopulations or perform latent class analysis to empirically determine the number of subpopulations to model. Given the goal of comparing latent variable mixture modeling to traditional linear modeling techniques, we selected the simplest latent variable mixture model for this initial assessment. We observed dramatic results, but increased latent variable flexibility could further improve AKI modeling. A second limitation was the small number of patients that developed moderate or severe AKI (100 or 200% ΔSCr – KDIGO stage II or III), which limited our power to compare latent variable mixture modeling to linear modeling techniques with high precision in patients with moderate or severe AKI. A majority of patients that develop postoperative AKI, however, develop mild AKI, and this outcome remains associated with major short and long-term morbidity [31–33]. Finally, it should be acknowledged that the predictive accuracy of the latent variable mixture model could be reduced in datasets missing model covariate information. However, due to increased model flexibility, the latent variable mixture model will always display equal to or better predictive accuracy compared to a linear model with the same covariates.

## Conclusions

A latent variable mixture model increased power to identify established AKI risk factors, more accurately ranked the severity of patients’ 48-h ΔSCr, and more accurately predicted 48-h postoperative ΔSCr compared to a linear model. Latent variable mixture modeling may improve clinicians’ ability to identify novel risk factors and advance the understanding of AKI pathophysiology. Employment of this technique could also advance preoperative AKI risk stratification and provide opportunities to further phenotype and target higher risk patient subpopulations with specific monitoring, preventative strategies, and treatments. Latent variable mixture modeling may provide a powerful technique to advance the study of AKI.

## Additional file

Additional file 1: Table S1. Characteristics of the estimated latent variable mixture model subpopulations. (DOCX 17 kb)

## Abbreviations

AKI: Acute kidney injury
BIC: Bayesian information criteria
BMI: Body mass index
eGFR: Estimated glomerular filtration rate
KDIGO: Kidney disease improving global outcomes
Q-Q: Quantile-quantile
ΔSCr: Change in serum creatinine concentration
