# Supplementary material for: Latent variable modeling improves AKI risk factor identification and AKI prediction compared to traditional methods
Source: BMC Nephrol. 2017 Feb 8;18:55. doi: 10.1186/s12882-017-0465-1 (PMC5299779; doi:10.1186/s12882-017-0465-1)
Supplement: Additional file 1: Table S1. — Characteristics of the estimated latent variable mixture model subpopulations. (DOCX 17 kb) [file 12882_2017_465_MOESM1_ESM.docx]

**Table S1**. Latent variable mixture model subpopulation characteristics. Binary characteristics are reported as n (%) and continuous characteristics as median (10^th^ percentile, 90^th^ percentile).

| Characteristic | Subpopulation 1 (n=80) | Subpopulation 2 (n=532) |  |
| --- | --- | --- | --- |
| Age, years | 70 (53, 81) | 66 (50, 81) |  |
| Female | 22 (27.5%) | 164 (30.1%) |  |
| African American | 6 (7.5%) | 20 (3.8%) |  |
| Body mass index, kg/m^2^ | 29 (23, 40) | 28 (22, 36) | |
| Medical history |  |  |  |
| Hypertension | 79 (98.8%) | 462 (86.8%) |  |
| Congestive heart failure | 46 (57.5%) | 195 (36.7%) |  |
| Left ventricular ejection fraction, % | 55 (30, 60) | 60 (35, 60) |  |
| Myocardial infarction | 15 (18.8%) | 95 (17.9%) |  |
| Prior cardiac surgery | 21 (26.3%) | 88 (16.5%) |  |
| Diabetes | 35 (43.8%) | 166 (31.2%) |  |
| Current smoking | 8 (10.0%) | 79 (14.8%) |  |
| Chronic obstructive pulmonary disease | 13 (16.3%) | 51 (9.6%) |  |
| Peripheral vascular disease | 29 (36.3%) | 140 (26.3%) |  |
| Preoperative medication use |  |  |  |
| Statin | 62 (77.5%) | 352 (66.2%) |  |
| ACE inhibitor | 29 (36.3%) | 162 (30.5%) |  |
| Baseline laboratory data |  |  |  |
| Creatinine, mg/dl | 1.21 (0.80, 1.92) | 1.00 (0.73, 1.51) |  |
| eGFR, ml/min/1.73 m^2^ | 52.73 (33.2, 85.2) | 74.6 (40.6, 98.0) |  |
| Hematocrit, % | 38 (30, 45) | 41 (33, 46) |  |
| Perioperative atorvastatin treatment assignment | 41 (51.3%) | 265 (49.8%) |  |
| Procedure characteristics |  |  |  |
| CABG surgery | 43 (53.8%) | 257 (48.3%) |  |
| Valve surgery | 50 (62.5%) | 344 (64.7%) |  |
| Cardiopulmonary bypass use | 59 (73.8%) | 373 (70.1%) |  |
| Cardiopulmonary bypass time, min | 114.5 (0.0, 214.9) | 110.0 (0.0, 210.0) |  |
| Aortic cross clamp use | 43 (53.8%) | 245 (46.1%) |  |
| Aortic cross clamp time, min | 58.0 (0.0, 153.2) | 0.0 (0.0, 136.9) |  |
| Intraoperative fluids |  |  |  |
| Intravenous crystalloid, mL | 1550 (1000, 2605) | 1600 (1000, 3000) |  |
| Intravenous hydroxyethyl starch, mL | 0 (0, 500) | 0 (0, 0) |  |
| Urine output, mL | 350 (149, 876) | 450 (186, 989) |  |
| Arterial lactate, max intraoperative, mmol/L | 1.7 (0.7, 3.8) | 1.7 (0.9, 3.7) |  |
| Length of surgery, hours | 5.4 (3.9, 7.8) | 5.1 (3.6, 7.8) |  |

eGFR, estimated glomerular filtration rate using CKD-Epi formula; CABG, coronary artery bypass grafting; max, maximum
